# Are Sex and Gender Dimensions Accounted for in NICE and SIGN Psychiatry Guidelines? A Systematic Review

**DOI:** 10.1192/bjo.2025.10137

**Published:** 2025-06-20

**Authors:** Rachel Hulme, Kate Womersley, Marina Politis, Alice Witt

**Affiliations:** ^1^The George Institute for Global Health, London, United Kingdom; ^2^Imperial College London, London, United Kingdom

## Abstract

**Aims:** Sex and gender are critical determinants in the diagnosis, progression, and management of psychiatric conditions, influencing disease epidemiology, symptom presentation, treatment responses, and access to care. However, the extent to which these factors are systematically incorporated into UK psychiatric clinical guidelines has been unclear. To date, no review has assessed how sex and gender considerations are addressed in guidelines produced by the National Institute for Health and Care Excellence (NICE) or the Scottish Intercollegiate Guidelines Network (SIGN).

This study aimed to evaluate the extent of sex and gender integration within psychiatric guidelines. It is the first to systematically assess these dimensions across NICE’s “*Mental health, behavioural, and neurodevelopmental conditions*” category and SIGN’s “*Mental health and behavioural conditions*” category, which encompass psychiatric and related conditions.

**Methods:** A systematic review of all NICE and SIGN psychiatry guidelines was conducted to assess how sex and gender considerations were incorporated across key areas: epidemiology, clinical presentation, investigations, and management. The gender composition of guideline committee chairs was also evaluated. Psychiatry guidelines were ranked relative to other medical specialties to determine their comparative performance.

**Results:** This review identified significant gaps in the integration of sex and gender considerations across NICE and SIGN psychiatry guidelines. Across NICE psychiatry guidelines, only 72% referenced sex and/or gender, and just 28% addressed these factors beyond reproductive contexts. While differential disease management (52%) and epidemiology (28%) were the most frequently considered aspects, investigations (17%) and clinical presentation (7%) were rarely discussed.

Psychiatry ranked second among NICE specialty categories for integrating sex and gender considerations, and scored second-best for women committee chair representation. This is significant because guidelines chaired by women tended to incorporate sex and gender considerations more comprehensively than those chaired by men. Results from SIGN psychiatry guidelines demonstrated similar trends.

Thematic analysis revealed that NICE and SIGN psychiatry guidelines were more likely than other specialties to acknowledge gendered challenges in accessing care, caregiving roles, social support networks, and current evidence gaps.

**Conclusion:** As a specialty in which both biological and social determinants are central to diagnosis and treatment, psychiatry is well-positioned to lead improvements in sex and gender-sensitive clinical guidance. Despite psychiatry’s relatively strong performance compared with other specialties, significant gaps remain, particularly in differentiated clinical presentations. NICE and SIGN must establish robust mechanisms to embed sex and gender disaggregated evidence into guidelines. Psychiatrists have a critical opportunity to drive improvements to enhance equity and patient outcomes.

